# The timeliness of health plan drug coverage policy changes to FDA label revisions

**DOI:** 10.1093/haschl/qxag034

**Published:** 2026-02-13

**Authors:** Daniel E Enright, Molly T Beinfeld, Claire H Brennan, Yichen Lin, James D Motyka, Jonathan D Campbell, James D Chambers

**Affiliations:** Center for the Evaluation of Value and Risk, Institute for Clinical Research and Health Policy Studies,Tufts Medical Center, Boston, MA 02111, United States; Center for the Evaluation of Value and Risk, Institute for Clinical Research and Health Policy Studies,Tufts Medical Center, Boston, MA 02111, United States; Center for the Evaluation of Value and Risk, Institute for Clinical Research and Health Policy Studies,Tufts Medical Center, Boston, MA 02111, United States; Center for the Evaluation of Value and Risk, Institute for Clinical Research and Health Policy Studies,Tufts Medical Center, Boston, MA 02111, United States; National Pharmaceutical Council, Washington, DC 20006, United States; National Pharmaceutical Council, Washington, DC 20006, United States; Center for the Evaluation of Value and Risk, Institute for Clinical Research and Health Policy Studies,Tufts Medical Center, Boston, MA 02111, United States

**Keywords:** FDA label revisions, health plan coverage policies, patient access to care, policy update timeliness, specialty drugs

## Abstract

**Introduction:**

Health plan coverage policies determine specialty drug access and should reflect evolving standards of care; however, the timeliness of policy updates remains unclear.

**Methods:**

We used the Tufts Medical Center Specialty Drug Evidence and Coverage (SPEC) Database to evaluate how quickly US commercial health plans updated coverage following Food and Drug Administration (FDA) label changes from 2019 to 2022. We calculated time from label revision to policy update using Kaplan–Meier methods, with new indications for existing drugs as a comparator, and applied Cox proportional hazards models to assess factors associated with update speed.

**Results:**

We identified 79 label expansions and 8 contractions across 1279 policies and excluded 421 already aligned with the revised label or using sufficiently broad language. Among 858 remaining decisions, the median time to policy update following label revisions was 29.7 weeks (95% CI: 26.9-33.6), compared to 13.4 weeks for new indications. Coverage updates were faster for label contractions vs expansions (hazard ratio [HR] = 1.30; *P* = 0.024), oncology vs non-oncology (HR = 1.82; *P* < 0.001), and self-administered vs physician-administered drugs (HR = 1.76; *P* < 0.001). We observed substantial variation across plans (15.1-55.4 weeks).

**Conclusion:**

Despite many policies already aligning with FDA label revisions, coverage updates were often slow and inconsistent. Improving timeliness may improve equitable access to care.

## Introduction

The US Food and Drug Administration (FDA) periodically updates drug labels to reflect new clinical evidence, safety data, or regulatory decisions. These updates ensure that approved indications align with a drug's appropriate use in current medical practice.^1^ FDA label changes may be particularly consequential for specialty therapies, which often treat serious or rare conditions with limited treatment options.^2,3^

Health plan coverage policies play a critical role in determining patient access to specialty therapies. To promote appropriate access, these policies should incorporate the latest clinical evidence and reflect current FDA-approved indications.^4-6^

While prior studies have explored aspects of specialty drug coverage, including variation in coverage criteria, alignment with clinical guidelines, and trends in utilization management over time,^7-9^ little is known about how promptly health plans revise their coverage criteria in response to FDA label changes. Lags could lead to inappropriate restrictions or delays in patient access.

This study addresses this gap by conducting 4 analyses. First, we determined the frequency with which US commercial health plans updated their specialty drug coverage policies within 2 years of an FDA label revision and used new indication approvals for existing therapies as a benchmark for comparison. Second, we used Kaplan–Meier (KM) survival curves to evaluate the timeliness of updates following label revisions compared with new indication approvals. Third, we assessed variation in update timing across health plans, treatment characteristics (eg, oncology or orphan status), direction of the label change (eg, expansion vs contraction of the eligible population), and type of revision (eg, age eligibility). Fourth, we examined factors associated with responsiveness—specifically, whether treatment characteristics and direction of label change were linked to policy update timing. To our knowledge, this is the first study to systematically evaluate how quickly health plans respond to FDA label changes across a wide range of specialty drugs.

## Methods

### Data sources

#### Coverage information and included drugs

We used the Tufts Medical Center Specialty Drug Evidence and Coverage (SPEC) Database, which contains publicly available coverage decisions for 469 drugs from 18 large US commercial health plans (Appendix 1) (To access the Appendix, click on the Details tab of the article). These plans represent approximately 70% of the commercially insured market, or roughly 200 million covered lives. For this analysis, we excluded coverage policies issued by one of the 18 plans in SPEC due to insufficient longitudinal data.

The SPEC Database has tracked coverage since August 2017 and organizes decisions at the drug-indication pair level. For example, since FDA approved dupilumab for both asthma and atopic dermatitis, SPEC includes separate coverage decisions for each indication.

At the time of this analysis, the database included 14 912 active coverage decisions corresponding to 1032 drug-indication pairs. Data were current as of August 2024 for this study. Additional details on the SPEC Database are available elsewhere.^8,10^

#### FDA label revisions

We identified FDA label revisions for drugs included in the SPEC database by reviewing the Drugs@FDA database^11^ for updates issued between January 2019 and August 2022. We reviewed each update to identify substantive changes to the indications and usage sections, focusing specifically on revisions that altered the eligible patient population. For example, in May 2020, the FDA expanded the label for dupilumab for atopic dermatitis to include patients aged 6 years and older, relaxing the previous lower bound on age of 12 years.

We only included label revisions identified as either expansions (broadened the approved patient population) or contractions (narrowed the population). We excluded updates unrelated to the approved indication, for instance, when the FDA added a black box warning to enfortumab vedotin-ejfv for locally advanced or metastatic urothelial cancer in July 2021. We also excluded revisions that exclusively standardized label language across similar therapies. A notable example occurred in 2019, when the FDA revised the labels of 12 multiple sclerosis treatments to specify use in clinically isolated syndrome, relapsing-remitting disease, and active secondary progressive disease. Previously, labels referred only to “relapsing forms of multiple sclerosis,” with some also noting use to decrease the frequency of clinical exacerbations, language that was removed during standardization.

We designated expansions as falling into one or more of the following 4 categories: (1) age-based expansions, such as the revised dupilumab indication that reduced the minimum age; (2) revised prior therapy requirements, such as when the FDA expanded pembrolizumab for classical Hodgkin's lymphoma from third line to second line use; (3) new drug combinations, such as the inclusion of daratumumab and dexamethasone with carfilzomib for treating multiple myeloma; and (4) new patient subgroup expansions, such as when the FDA extended ribociclib's indication for breast cancer to include male patients. We allowed for the designation of multiple categories because some label updates involved multiple expansion types. For example, the FDA revised adalimumab's indication for ulcerative colitis by both lowering the eligible age to 5 years and removing a prior therapy requirement.

We classified label contractions into 2 categories: (1) changes to prior therapy requirements that restricted eligibility (eg, the revised upadacitinib indication for rheumatoid arthritis, which now requires inadequate response or intolerance to TNF blockers rather than methotrexate) and (2) narrowing of eligible patient subgroups based on clinical characteristics (eg, the revised pembrolizumab indication for adjuvant melanoma, which limited treatment to individuals with stage IIB, IIC, or III disease).

To provide a comparator for FDA label revisions, we identified approvals of entirely new indications for existing therapies between 2019 and 2022, such as the May 2021 approval of ozanimod for ulcerative colitis.

Two researchers independently reviewed SPEC Database coverage decisions for drug-indication pairs affected by FDA label revisions to determine when plans updated their policies. A third researcher verified whether the updated coverage aligned with the revised FDA label. When a revised label included multiple changes—such as adalimumab's updated indication, which lowered the minimum age and removed a prior therapy requirement—we included only policies that addressed the full scope of the revision.

Researchers also identified the first coverage decision cataloged in the SPEC Database for each newly FDA-approved indication for drugs already on the market for another FDA-approved use.

For our time-to-event analyses, we excluded policies that deferred entirely to the FDA label (eg, “coverage is provided when used in accordance with FDA labeling for oncologic indications”) and those already aligned with the revised label at the time of the FDA update. For age-based revisions, we also excluded policies that lacked specific age criteria or used broad terms (eg, “FDA-approved age”) that encompassed the revised threshold.

We recorded the date of each updated coverage decision and calculated the time from FDA label revision to policy update. When multiple documents supported a single coverage decision, we used the earliest document issued after the label revision or, for entirely new indications, the date of approval. For one plan that does not date its policies, we used the first day of the month the document was added to the SPEC Database.

### Analyses

We conducted 4 analyses. In analysis 1, we assessed the frequency with which health plans updated coverage policies within 2 years of an FDA label revision and compared with policy issuance within 2 years of new indication approvals. We examined whether response rates varied by direction of revision (expansion vs contraction), treatment characteristics (eg, oncology or orphan status), and across health plans.

In analysis 2, we used KM survival curves to evaluate the timeliness of coverage updates following FDA label revisions compared with new indication approvals. For label revisions, the event of interest was issuance of a policy aligned with the revised FDA label; for new indications, it was issuance of a policy reflecting the new approval. For both groups, observations were censored at 2 years if no update occurred, consistent with prior research showing that most policy updates occur within that timeframe.^12^

In analysis 3, we used KM curves to examine differences in response times following FDA label revisions by revision direction, treatment characteristics, revision type, and health plan.

In analysis 4, we applied Cox proportional hazards regression to identify factors associated with the speed of policy updates following FDA label revisions. The model included revision direction, oncology status, orphan designation, and route of administration (self-administered vs physician-administered). We assessed the proportional hazards assumption using Schoenfeld residuals.

Finally, we conducted 2 sensitivity analyses focused on coverage policy issuance following FDA label revisions. First, when multiple documents supported a single decision, we measured response time using the most recent coverage document—rather than the earliest. Second, we excluded policies from the one health plan that does not date its coverage documents.

We used Stata 18.0 SE for all analyses.

Our study has limitations. First, while the SPEC Database captures publicly available policies, it may not reflect informal or interim access pathways that plans offer in practice that are not publicly available. Second, we did not evaluate the role of appeals processes or how often patients successfully gain access through appeals. It is likely that, in cases where a patient meets the criteria based on an FDA label change, plans may be inclined to grant access, even in the absence of a formally updated policy. Third, our findings may not be applicable to other types of payers (eg, Medicare, Medicaid) not in the SPEC Database.

## Results

We identified 87 FDA label revisions issued between 2019 and 2022 (Table 1), corresponding to 1479 potential coverage decisions. Of these, 200 drug–indication–plan pairs lacked a publicly available policy, leaving 1279 coverage decisions in our dataset. Of these, 160 (12.5%) decisions deferred to the FDA label, 128 (10.0%) were already aligned with the revised label at the time of the update, and 133 (10.4%) did not impose age restrictions or used broad language that encompassed the revised age threshold. As noted above, we excluded these decisions from our time-to-event analyses, as they lacked a measurable response time.

**Table 1.** Summary of identified label revisions by year.

	Total 2019-2022	2019	2020	2021	2022
**Total # revisions**	87	16	22	39	10
**Expansions *n* (%)**	79 (90.8%)	15 (93.8%)	22 (100%)	33 (84.6%)	9 (90.0%)
**Contractions *n* (%)**	8 (9.2%)	1 (6.2%)	0 (0.0%)	6 (15.4%)	1 (10.0%)
**Age revision *n* (%)**	31 (35.6%)	8 (50.0%)	8 (36.4%)	13 (33.3%)	2 (20.0%)
**Line of therapy revision *n* (%)**	17 (19.5%)	3 (18.8%)	5 (22.7%)	7 (18.0%)	2 (20.0%)
**New drug combination revision *n* (%)**	22 (25.3%)	4 (25.0%)	7 (31.8%)	9 (23.1%)	2 (20.0%)
**Subgroup revision *n* (%)**	12 (13.8%)	1 (6.3%)	1 (4.5%)	8 (20.5%)	2 (20.0%)
**Multiple revision types *n* (%)**	5 (5.7%)	0 (0.0%)	1(4.5%)	2 (5.1%)	2 (20.0%)
**Cancer *n* (%)**	44 (50.6%)	4 (25.0%)	11 (50.0%)	22 (56.4%)	7 (70.0%)
**Orphan n (%)**	40 (46.0%)	10 (62.5%)	8 (36.4%)	16 (41.0%)	6 (60.0%)

Source: Author's analysis of specialty drugs contained in the Tufts Medical Center's Specialty Drug Evidence and Coverage (SPEC) database and drug labels gathered from Drugs@FDA.^11^

We identified 104 new indications added to FDA labels between 2019 and 2022, corresponding to 1768 potential coverage decisions. Of these, 231 drug–indication–plan pairs lacked a publicly available policy, leaving 1537 coverage decisions. Among these, 282 already covered the newly approved indication; most (60.6%) were oncology decisions deferring to FDA labeling, while the remainder covered prior off-label use. The remaining 1255 decisions were included in the analysis.

Analysis 1: Of the remaining 858 coverage decisions (Appendix 2) (To access the Appendix, click on the Details tab of the article), 671 (78.2%) responded to FDA label revisions within 2 years, compared to 1217 of 1255 (97.0%) of decisions for new indications issued within 2 years of approval. For FDA label revisions, response rates were higher for label contractions compared to expansions (90.5% vs 76.7%), for oncology than non-oncology revisions (86.6% vs 69.5%), and for orphan treatments than non-orphan treatments (82.3% vs 74.8%). Response rates to FDA label revisions also varied across plans, ranging from 60.0% to 87.1% (Table 2).

**Table 2.** Median response time to FDA label revisions by health plan.

Health plan	Total # coverage decisions	# Revised decisions n (%)	Median response time (weeks)^a^
All health plans	858	671 (78.2%)	29.7 (95% CI 26.9-33.6)
Health plan 1	68	57 (83.8%)	34.1 (95% CI 21.4-39.0)
Health plan 2	67	51 (76.1%)	15.1 (95% CI 11.9-55.0)
Health plan 3	66	49 (74.2%)	27 (95% CI 19.1-34.6)
Health plan 4	62	43 (69.4%)	46 (95% CI 37.4-60.9)
Health plan 5	61	51 (83.6%)	18 (95% CI 14.9-24.1)
Health plan 6	60	49 (81.7%)	47.9 (95% CI 31.1-61.4)
Health plan 7	56	44 (78.6%)	34.4 (95% CI 27.6-49.6)
Health plan 8	55	47 (85.5%)	24.3 (95% CI 13.1-35.6)
Health plan 9	52	39 (75.0%)	24.3 (95% CI 13.3-47.0)
Health plan 10	46	40 (87.0%)	29.9 (95% CI 25.9-36.7)
Health plan 11	45	38 (84.4%)	15.7 (95% CI 13.4-26.9)
Health plan 12	44	29 (65.9%)	55.4 (95% CI 29.7-92.0)
Health plan 13	43	33 (76.7%)	16.1 (95% CI 10.9-19.3)
Health plan 14	40	24 (60.0%)	53 (95% CI 17.1-^b^)
Health plan 15	37	32 (86.5%)	15.9 (95% CI 9.6-55.0)
Health plan 16	31	27 (87.1%)	49.4 (95% CI 26.3-63.3)
Health plan 17	25	18 (72.0%)	21.4 (95% CI 18.1-71.4)

Source: Author's analysis of specialty drugs contained in the Tufts Medical Center's Specialty Drug Evidence and Coverage (SPEC) database and drug labels gathered from Drugs@FDA.^11^

^a^Kaplan–Meier adjusted.

^b^For one plan, the upper bound of the 95% CI for the median response time could not be estimated due to substantial right-censoring, which limited the ability to precisely determine the upper range of the survival distribution.

Analysis 2: KM analysis showed that the median time to policy update following FDA label revisions was 29.7 weeks (95% CI 26.9-33.6), compared with 13.4 weeks (95% CI 12.7-14.3) for coverage issuance after approval of new indications.

**Figure 1.**
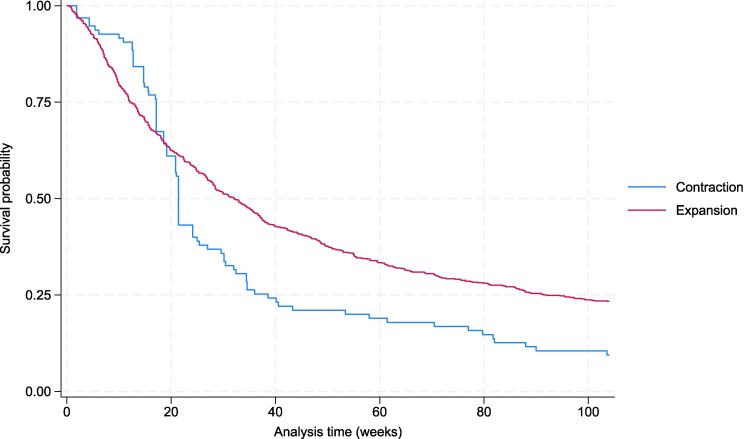
Kaplan–Meier survival curves—health plan response time to FDA label expansions vs contractions. Source: Author's analysis of specialty drugs contained in the Tufts Medical Center's Specialty Drug Evidence and Coverage (SPEC) database and drug labels gathered from Drugs@FDA.^11^

Analysis 3: For FDA label revisions, median response time was shorter for label contractions than expansions (21.4 vs 32.1 weeks) (Figure 1), for oncology treatments compared to non-oncology (28.6 vs 31.0 weeks) (Appendix 3) and for orphan treatments compared to non-orphan (28.7 vs 30.1 weeks) (Appendix 4) (To access the Appendix, click on the Details tab of the article).

Among label expansions, response times varied by revision type. Median response time for age-based revisions was faster than for other revision types (27.6 vs 36.3 weeks). A similar pattern held for revisions involving new drug combinations (27.6 vs 35.1 weeks). In contrast, response to revisions involving patient subgroups was slower (42.3 vs 28.7 weeks), as was response to revisions involving prior therapy requirements. For prior therapy requirement revisions, fewer than half of the relevant coverage decisions were updated, so a median response time could not be calculated. We did not assess response time by revision type for coverage decisions responding to label contractions, as most (87.4%) were for prior therapy requirements.

Response time varied substantially across individual health plans, with the fastest-responding plan having a median response time of 15.1 weeks, and the slowest-responding plan a median of 55.4 weeks (Table 2).

Analysis 4: Univariable Cox regression analyses found that oncology status (HR = 1.40; *P* < 0.01), orphan designation (HR = 1.22; *P* < 0.01), self-administered treatments (HR = 1.26; *P* < 0.01), and label contractions (HR = 1.42; *P* < 0.01) were associated with faster response times. In multivariable analysis, oncology status (HR = 1.82), self-administration (HR = 1.76), and label contractions (HR = 1.30) remained significantly associated with quicker response (*P* < 0.05) (We assessed proportional hazards assumption using Schoenfeld residuals and found oncology status to violate PH assumption (*P* < 0.001). To address this, we included a time-dependent covariate for oncology status in our model. In the updated model, label contractions remained associated with quicker responses (HR = 1.32; *P* < 0.01), while oncology status was associated with a slower response (HR = 0.50; *P* = 0.005). However, this effect diminished over time, as indicated by a significant time-varying interaction (HR = 1.40; *P* < 0.001)). Orphan designation remained associated with faster response but was no longer statistically significant (Table 3).

**Table 3.** Cox proportional hazards regression results for health plan response to FDA label revisions—univariable and multivariable.

Variable	# Coverage decisions	Univariable		Multivariable	
		HR (95% CI)	*P*-value	HR (95% CI)	*P*-value
**Revision Direction**					
Expansion	763	Reference		Reference	
Contraction	95	1.42 (1.13-1.78)	0.003	1.30 (1.03-1.63)	0.024
**Oncology status**					
Non-oncology	419	Reference		Reference	
Oncology	439	1.40 (1.20-1.63)	0.000	1.82 (1.50-2.21)	0.000
**Orphan status**					
Non-orphan	473	Reference		Reference	
Orphan	385	1.22 (1.05-1.42)	0.010	1.09 (0.93-1.28)	0.312
**Treatment administration**					
Physician-administered	553	Reference		Reference	
Self-administered	305	1.26 (1.08-1.47)	0.004	1.76 (1.45-2.13)	0.000

Source: Author's analysis of specialty drugs contained in the Tufts Medical Center's Specialty Drug Evidence and Coverage (SPEC) database and drug labels gathered from Drugs@FDA.^11^

Sensitivity analysis: Measuring response time based on the most recent document issued after the label revision yielded a median response 31.3 weeks, compared to 29.7 when measuring response time using the first document issued following the label revision. Analysis excluding the one health plan that does not date its coverage documents yielded a median response time of 29.1 weeks.

## Discussion

Nearly one third of coverage policies were already aligned with revised FDA labels, indicating that in these instances the plans' criteria were sufficiently adaptable. Among those requiring revision, the median lag exceeded 6 months underscoring wide variation in responsiveness. Because label revisions often expand or refine appropriate use, delayed updates may limit timely patient access or prolong coverage for outdated indications.

This gap between regulatory action and payer response highlights an important yet underexamined aspect of the US healthcare system: the speed at which health plans incorporate FDA-approved changes into their coverage policies. Given that FDA label revisions often expand access to therapies or clarify appropriate use, delayed payer responsiveness may create avoidable barriers to care. Conversely, slow responses to label contractions may expose patients to treatments no longer aligned with current safety and efficacy standards.

Although most plans eventually updated coverage, we found the frequency and timeliness of response varied widely across health plans, therapeutic areas, route of administration, and types of label changes. These differences may have important implications: patients' access to therapies based on the most current FDA label information can depend on their health plan. Consequently, physicians may need to tailor treatment decisions not only to clinical needs but also to plan-specific coverage criteria. The variation may stem from differences in plan resources or internal processes. For instance, some plans follow regular policy update schedules, such as annual reviews, while others may revise coverage more frequently or on a less formal or rolling basis.

We found that health plans responded more frequently and more quickly to approvals of entirely new indications than to revisions of existing ones. Although new indications may represent more substantial changes that warrant rapid response, this pattern suggests opportunities for more timely updates following label revisions.

We also found that plans responded more quickly to label contractions than expansions (median 21.4 vs 32.1 weeks). This pattern may reflect heightened attention to safety-related concerns or a greater perceived need to prevent inappropriate utilization. Budgetary considerations may also play a role, as aligning with label contractions typically reduces the number of eligible patients, thereby lowering utilization and costs. In contrast, delayed responses to label expansions may result in newly eligible patients experiencing prolonged access barriers. Further, we found that oncology status and, in unadjusted models, orphan status, were associated with more rapid health plan response, possibly reflecting greater perceived clinical urgency or plan oversight in these areas.

Of the coverage decisions we reviewed, we excluded just under a third from our analysis, either because they deferred to the FDA label (for the entirety of coverage or age of eligibility), were already aligned with the revised label at the time of the revision, or, for age eligibility revisions, did not impose age limits. This highlights that some plans integrate FDA labeling more directly into their frameworks than others. Notably, most of the decisions excluded due to deferral to the FDA label came from 4 health plans, suggesting that access in these plans may align with FDA label changes more quickly than in others.

Our findings underscore the distinct roles of the FDA and health plans. While the FDA bases label changes on clinical trial data, health plans consider medical necessity and cost, often incorporating real-world evidence, clinical guidelines, and health technology assessments.^13,14^ As a result, plans may retain existing criteria despite FDA updates. For instance, although the FDA removed the requirement for failure of immunosuppressants such as corticosteroids, azathioprine or 6-mercaptopurine (6-MP) before initiating adalimumab for ulcerative colitis, many plans were slow to revise their policies—likely viewing this step as clinically appropriate and cost-effective. Notably, updates were slowest and least frequent for label expansions involving prior therapy requirements, while plans responded more promptly to changes in age limits and combination therapy use.

Our findings raise the possibility that plans may also be slow to respond to other key milestone events, including updates to clinical guidelines, or the emergence of new clinical evidence. Future research should evaluate how responsive plans are to these types of changes, which are equally critical for ensuring that coverage policies reflect current best practices.

Moving forward, improving the timeliness of coverage updates may help ensure that patients receive appropriate, up-to-date care consistent with the latest clinical and regulatory evidence. Policymakers and other stakeholders could consider mechanisms to encourage greater transparency and accountability in how and when plans incorporate FDA label changes.^15^ For example, organizations such as the Academy of Managed Care Pharmacy and America's Health Insurance Plans could promote best practices for timely and transparent policy updates, while groups like the National Committee for Quality Assurance could incorporate measures of timeliness and transparency into their commercial health plan ratings.^16^

Future research could explore the downstream consequences of delayed coverage alignment, such as impacts on prescribing, utilization, and patient outcomes, as well as potential solutions to improve policy responsiveness.

## Conclusion

Commercial health plans vary in the timeliness of updating coverage policies following FDA label revisions. These delays may limit access to effective therapies, particularly for FDA label expansions, and contribute to inconsistency in access across plans. Ensuring timely alignment between regulatory decisions and payer policies is important for improving equitable and evidence-based access to specialty treatments.

## Supplementary Material

qxag034_Supplementary_Data
