# Supplementary material for: The timeliness of health plan drug coverage policy changes to FDA label revisions
Source: Health Aff Sch. 2026 Feb 13;4(2):qxag034. doi: 10.1093/haschl/qxag034 (PMC12927503; doi:10.1093/haschl/qxag034)

Appendix 1: U.S. commercial health plans included in the SPEC database

- Aetna
- Anthem
- BCBS Florida
- BCBS Massachusetts
- BCBS Michigan
- BCBS New Jersey
- BCBS North Carolina
- BCBS Tennessee
- CareFirst BCBS
- Centene
- Cigna
- EmblemHealth
- Health Care Service Corporation (HCSC; comprised of BCBS Plans in Illinois, Montana, New Mexico, Oklahoma, and Texas)
- Highmark
- Humana
- Independence BC
- Kaiser Permanente
- UnitedHealthcare

Note: Health plans in SPEC collectively cover 200 million lives, representing 70% of the commercially insured population. Source: National Association of Insurance Commissioners (NAIC) 2023 Accident and Health Policy Experience Report. Available at: <https://content.naic.org/sites/default/files/publication-ahp-lr-accident-health-report.pdf>

Appendix 2: Summary of coverage decisions included for analysis

| Total # decisions | 858 |
| --- | --- |
| Expansions n (%) | 763 (88.9%) |
| Contractions n (%) | 95 (11.1%) |
| Age revision n (%) | 272 (31.7%) |
| Line of therapy revision n (%) | 176 (20.5%) |
| New drug combination revision n (%) | 233 (26.0%) |
| Subgroup revision n (%) | 134 (15.6%) |
| Multiple revision types n (%) | 53 (6.2%) |
| Cancer n (%) | 439 (51.2%) |
| Orphan n (%) | 358 (44.9%) |

Appendix 3: Kaplan-Meier survival curves – health plan response time to FDA label changes for oncology vs. non-oncology therapies


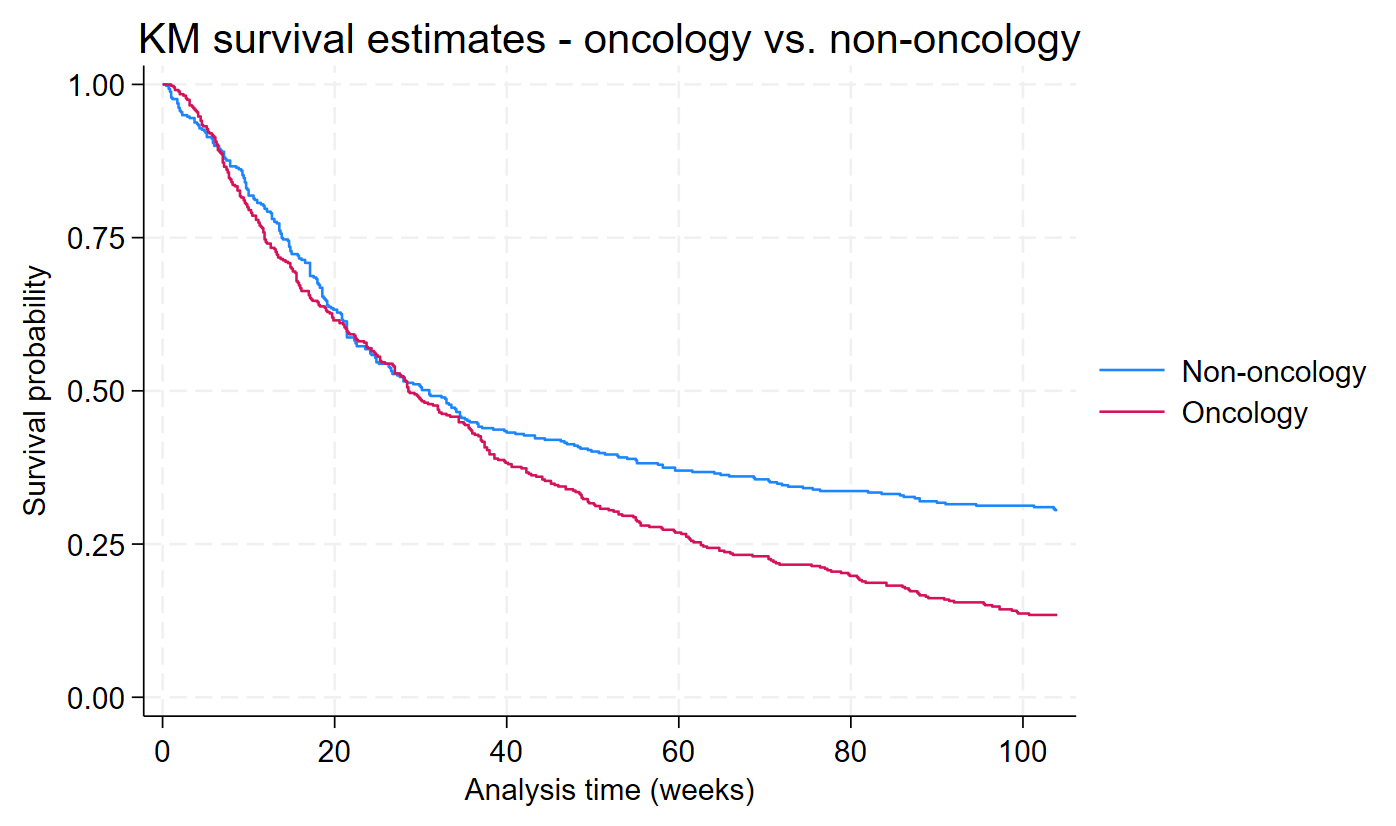


Appendix 4. Kaplan-Meier survival curves – health plan response to FDA label changes for orphan vs. non-orphan therapies


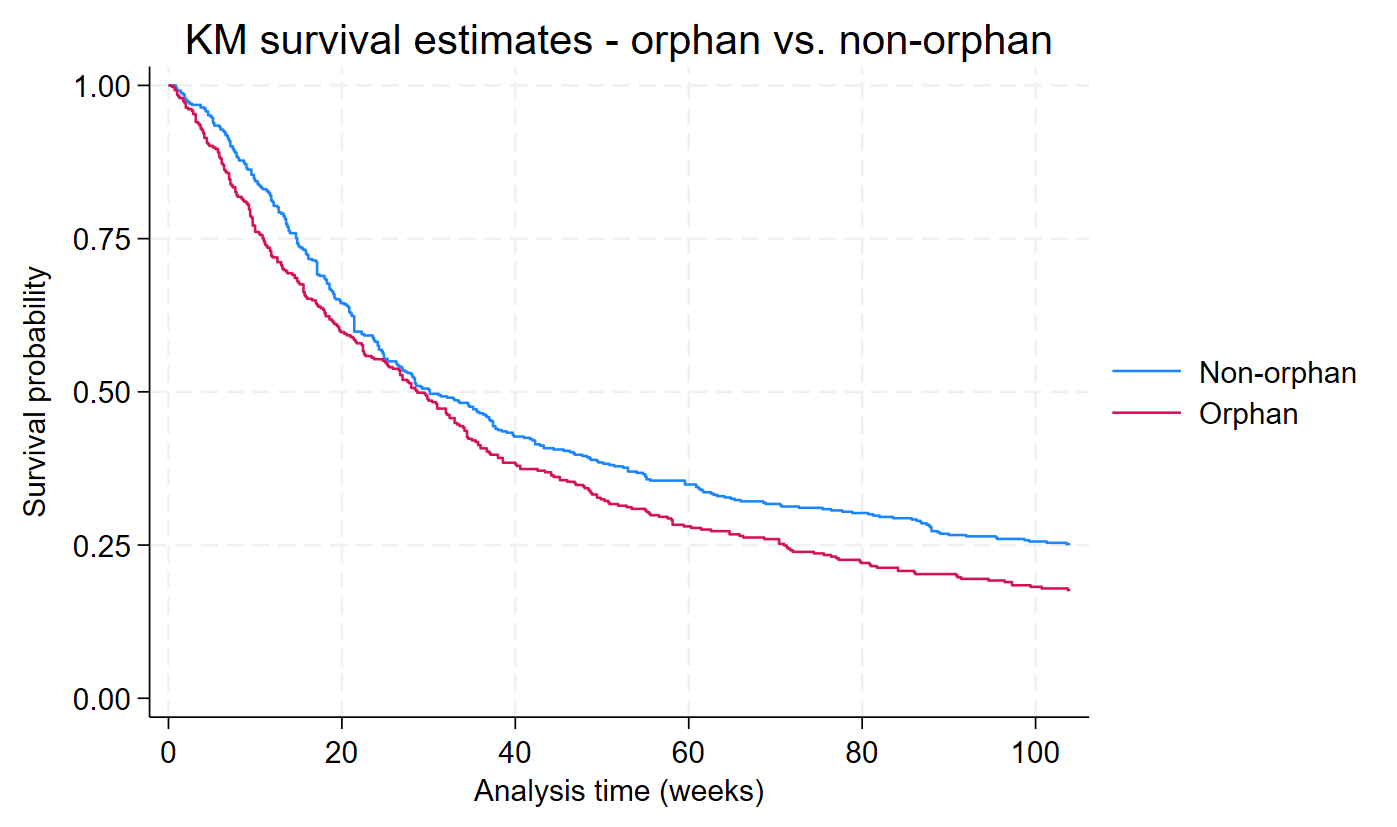

Supplement: qxag034_Supplementary_Data [file qxag034_supplementary_data.zip › Appendix Material.docx]
